# Effectiveness of JYNNEOS Vaccine Against Diagnosed Mpox Infection — New York, 2022

**DOI:** 10.15585/mmwr.mm7220a4

**Published:** 2023-05-19

**Authors:** Eli S. Rosenberg, Vajeera Dorabawila, Rachel Hart-Malloy, Bridget J. Anderson, Wilson Miranda, Travis O’Donnell, Charles J. Gonzalez, Meaghan Abrego, Charlotte DelBarba, Cori J. Tice, Claire McGarry, Ethan C. Mitchell, Michele Boulais, Bryon Backenson, Michael Kharfen, James McDonald, Ursula E. Bauer

**Affiliations:** ^1^New York State Department of Health; ^2^Department of Epidemiology and Biostatistics, School of Public Health, University at Albany, Rensselaer, New York; ^3^Center for Collaborative HIV Research in Practice and Policy, School of Public Health, University at Albany, Rensselaer, New York.

In 2022, an international *Monkeypox* *virus* outbreak, characterized by transmission primarily through sexual contact among gay, bisexual, and other men who have sex with men (MSM), resulted in 375 monkeypox (mpox) cases in the state of New York outside of New York City (NYC).*^,^^†^ The JYNNEOS vaccine (Modified Vaccinia Ankara vaccine, Bavarian Nordic), licensed by the U.S. Food and Drug Administration (FDA) against mpox as a 2-dose series, with doses administered 4 weeks apart,^§^ was deployed in a national vaccination campaign.^¶^ Before this outbreak, evidence to support vaccine effectiveness (VE) against mpox was based on human immunologic and animal challenge studies (*1*–*3*). New York State Department of Health (NYSDOH) conducted a case-control study to estimate JYNNEOS VE against diagnosed mpox in New York residents outside of NYC, using data from systematic surveillance reporting. A case-patient was defined as a man aged ≥18 years who received a diagnosis of mpox during July 24–October 31, 2022. Contemporaneous control patients were men aged ≥18 years with diagnosed rectal gonorrhea or primary syphilis and a history of male-to-male sexual contact, without mpox. Case-patients and control patients were matched to records in state immunization systems. JYNNEOS VE was estimated as 1 – odds ratio (OR) x 100, and JYNNEOS vaccination status (vaccinated versus unvaccinated) at the time of diagnosis was compared, using conditional logistic regression models that adjusted for week of diagnosis, region, patient age, and patient race and ethnicity. Among 252 eligible mpox case-patients and 255 control patients, the adjusted VE of 1 dose (received ≥14 days earlier) or 2 doses combined was 75.7% (95% CI = 48.5%–88.5%); the VE for 1 dose was 68.1% (95% CI = 24.9%–86.5%) and for 2 doses was 88.5% (95% CI = 44.1%–97.6%). These findings support recommended 2-dose JYNNEOS vaccination consistent with CDC and NYSDOH guidance.

The first mpox case in New York outside NYC was reported on June 2, 2022. On June 28, the U.S. Department of Health and Human Services’ Administration for Strategic Preparedness and Response announced a phased, jurisdictional rollout of the JYNNEOS vaccine from the Strategic National Stockpile, prioritizing postexposure prophylaxis (PEP) and vaccination of persons with recent or ongoing risks for mpox infection.** The first New York allocation of 2,206 vials was received July 6. By September 12, a total of 35,666 vials had been delivered.^††^ NYSDOH coordinated vaccine distribution in New York outside NYC with local health departments and community organizations.

All mpox, gonorrhea, and syphilis diagnoses in New York outside of NYC are reportable to NYSDOH.^§§^ Reports are investigated by public health staff members and entered into the Communicable Disease Electronic Surveillance System (CDESS). Case-patients were males at birth aged ≥18 years with a diagnosis of laboratory-confirmed mpox from whom specimens were collected during July 24–October 31, 2022 (2 weeks after vaccine campaign launch through the end of mandatory dose reporting to the New York State Immunization Information System [NYSIIS]). Control patients were males at birth aged ≥18 years with rectal gonorrhea or primary syphilis diagnosed within the same time frame as the mpox cases, and with presumptive sexual contact with a male or transgender person.^¶¶^

Demographic characteristics of case- and control patients were compared using Wilcoxon rank-sum and Pearson’s chi-square tests. Case- and control patient records in CDESS were matched to NYSIIS*** by name and date of birth to ascertain JYNNEOS vaccination status and history, an approach similar to that used in a COVID-19 VE study (*4*). Vaccination status was categorized into four groups, including one unvaccinated group (no JYNNEOS doses received) or one of three vaccinated groups: 1) with mpox or sexually transmitted infection (STI) specimen collected <14 days after receipt of dose 1; 2) ≥14 days after dose 1; or 3) after dose 2 (*5*,*6*). To estimate adjusted VE, conditional logistic regression models of case- and control patient vaccination status and dose history were used, matched on diagnosis week, with covariates including age, race and ethnicity, and region within New York outside NYC. VE values (with 95% CIs) were estimated as 1 – OR x 100, comparing each vaccinated category with the unvaccinated group.^†††^ Four sensitivity analyses were conducted to examine uncertainties in case- and control patient definitions. All 1-dose VE estimates were reported for doses received ≥14 days earlier, unless otherwise specified. Statistical analyses were carried out using SAS software (version 9.4; SAS Institute). This analysis was determined to be nonresearch by the NYSDOH Institutional Review Board.

During June 2–December 31, 2022, a total of 375 mpox cases were reported to NYSDOH and the administration of 27,385 JYNNEOS doses was recorded in NYSIIS, including 16,769 (61%) first doses and 10,616 (39%) second doses (Figure). The reported number of cases peaked in mid-August, 5 weeks after launch of the JYNNEOS vaccination campaign. During July 24–October 31, a total of 252 male mpox case-patients and 255 STI control patients (175 with rectal gonorrhea and 80 with primary syphilis) met inclusion criteria. The age distribution was similar for case-patients (median = 32.1 years; range = 18.5–66.4 years) and control patients (median = 31.3 years; range = 19.4–74.3 years) (p = 0.47). Among persons with known ethnicity, Hispanic ethnicity was more prevalent among case-patients (43.6%) than among controls (18.9%; p<0.001) (Table 1). In addition, 68.7% of case-patients lived in the metropolitan region outside NYC, compared with 35.7% of control patients (p<0.001).

**FIGURE:**
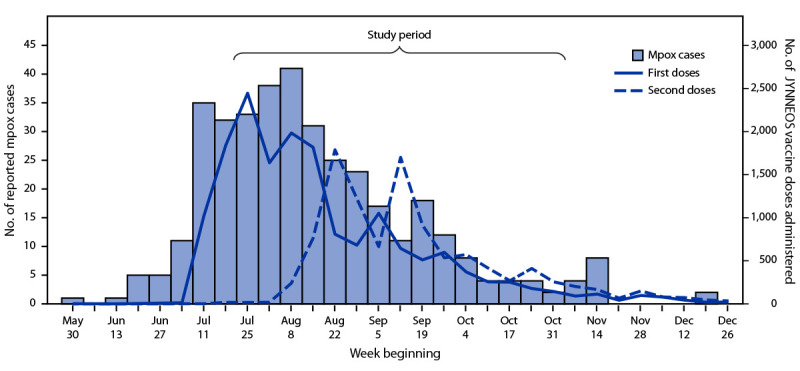
Reported mpox cases and first and second JYNNEOS vaccine doses administered, by week — New York,* June 2–December 31, 2022 **Abbreviation:** Mpox = monkeypox. * Outside of New York City.

**TABLE 1:** Demographic characteristics of case-patients with mpox and control patients with sexually transmitted infections* — New York,^†^ July 24, 2022–October 31, 2022

Characteristic	No. (%)		p-value
	Mpox case-patients (n = 252)	STI control patients* (n = 255)	
**Age group, yrs**			
18–29	94 (37.3)	111 (43.5)	0.34
30–39	90 (35.7)	75 (29.4)	
40–49	37 (14.7)	33 (12.9)	
≥50	31 (12.3)	36 (14.1)	
**Race and ethnicity** ^§^			
Black or African American, NH	48 (19.8)	68 (32.1)	<0.001
White, NH	69 (28.4)	90 (42.5)	
Hispanic or Latino	106 (43.6)	40 (18.9)	
Other, NH	20 (8.2)	14 (6.6)	
Unknown	9 (3.6)	43 (16.7)	
**Region**			
Metropolitan region outside NYC^¶^	173 (68.7)	91 (35.7)	<0.001
Rest of New York outside NYC	79 (31.3)	164 (64.3)	

**Abbreviations**: Mpox = monkeypox; NH = non-Hispanic; NYC = New York City; STI = sexually transmitted infection.

* Men with diagnosed rectal gonorrhea or primary syphilis and a history of male-to-male sexual contact.

^†^ Outside of New York City.

^§^ For race and ethnicity, the percentages and chi-square p-values are among case-patients and control patients for whom race and ethnicity were known.

^¶^ Includes Nassau, Putnam, Rockland, Suffolk, and Westchester counties.

Among the 252 mpox case-patients, 22 (8.7%) had received the JYNNEOS vaccine, including 10 (4.0%) who had received 1 dose <14 days earlier, 10 (4.0%) who had received 1 dose ≥14 days earlier, and two (0.8%) who had received 2 doses; 230 (91.3%) were not vaccinated (Table 2). Among 255 control patients, 51 (20%) had received the JYNNEOS vaccine, including 42 (16.5%) who received an STI diagnosis ≥14 days after receiving 1 dose (23; 9.0%) or 2 doses (19; 7.5%). This corresponded to adjusted VE for combined 1 dose or 2 doses of 75.7% (95% CI = 48.5%–88.5%); 1-dose VE was 68.1% (95% CI = 24.9%–86.5%) and 2-dose VE was 88.5% (95% CI = 44.1%–97.6%). No significant VE was observed within 13 days of receipt of dose 1. 

**TABLE 2:** JYNNEOS vaccination history and estimated vaccine effectiveness among case-patients with mpox and control patients with sexually transmitted infections — New York,* July 24, 2022–October 31, 2022

Vaccination status	Mpox case-patients (n = 252)	All STI controls (n = 255)	
	No. (%)	No. (%)	VE (95% CI)
Unvaccinated	230 (91.3)	204 (80.0)	Ref
0–13 days after first dose	10 (4.0)	9 (3.5)	–36.2 (<–100 to 56.3)
≥14 days after first dose	10 (4.0)	23 (9.0)	68.1 (24.9 to 86.5)
≥0 days after second dose	2 (0.8)	19 (7.5)	88.5 (44.1 to 97.6)
≥14 days after first dose or ≥0 days after second dose	12 (4.8)	42 (16.5)	75.7 (48.5 to 88.5)

**Abbreviations:** Mpox = monkeypox; Ref = referent group; STI = sexually transmitted infection; VE = vaccine effectiveness.

* Outside of New York City.

The first of the four sensitivity analyses (Supplementary Table, https://stacks.cdc.gov/view/cdc/128142) excluded men aged ≥50 years, who might have received a smallpox vaccine before routine nonmilitary U.S. vaccination ended in 1972; this analysis detected nearly identical VE as the main sensitivity analysis. The second sensitivity analysis included 71 secondary syphilis diagnoses in the control group, resulting in 1-dose or 2-dose combined VE of 64.8% (95% CI = 26.7%–83.1%). Among control patients, 213 (83.5%) had known reasons for testing: 88 (41.3%) because of symptoms, 19 (8.9%) because of partner referral, 103 (48.4%) for screening, and three (1.4%) for another reason. The third analysis restricted control patients to those persons testing for symptoms or referrals; 1-dose or 2-dose VE was 63.6% (95% CI = 8.0%–85.6%).^§§§^ The final sensitivity analysis limited observations to persons with known race and ethnicity and estimates increased modestly from the primary analysis, with 1-dose or 2-dose VE of 80.5% (95% CI = 56.1%–91.3%).

## Discussion

Receipt of 1 or 2 JYNNEOS doses was effective in preventing diagnosed mpox infection, with higher 2-dose VE of >88%. These findings support the approved use of the JYNNEOS vaccine as a 2-dose series for mpox prevention and, amid ongoing sexually related transmission of mpox, incorporating the JYNNEOS vaccine into a broader program of sexual health services.

Before this outbreak, evidence to support VE against mpox was based on human immunologic and animal challenge studies (*1*–*3*). Since the outbreak began, new estimates have been generated. CDC used multi-jurisdictional data on mpox patient vaccination status to estimate 9.6- and 7.4-fold incidence for unvaccinated at-risk males compared with 2-dose and 1-dose recipients, respectively (*5*,*6*). A similar United Kingdom analysis found 78% 1-dose VE,^¶¶¶^ and an Israeli cohort study found 86% 1-dose VE (*7*). These cohort studies are subject to biases; at-risk unvaccinated population estimates are uncertain and afford limited ability to control for confounding variables. For example, incidence among vaccinated persons might be reduced by persons with lower risk behaviors seeking vaccination, inflating incidence risk ratios and VE estimates. Case-control studies such as this one and others (*8*) can build in control for both risk factors and test-seeking, which was achieved in this study by sampling persons with diagnosed infections.

VE was moderately high according to the results of all sensitivity analyses. Lower VE observed when including secondary syphilis might reflect control patients with more remote risk behaviors or different clinical presentation. Lower VE when limiting control patients to persons who had testing because of symptoms or partner referral could reflect intended removal of persons at lower risk seeking health care or inadvertent removal of persons at higher risk accessing frequent screening.

No protection was present for 1 dose received <14 days earlier; however, this interval could include both persons who received PEP and those who were exposed after vaccination but before a protective immune response might be anticipated. Additional studies are needed to resolve these scenarios, with control groups better selected for studying PEP.

The findings in this report are subject to at least six limitations. First, uncontrolled confounding might remain, which could lead to under- or overestimation of VE. For example, some factors might positively link persons more likely to acquire mpox and receive the JYNNEOS vaccine, compared with the overall population of persons who acquire an STI. These factors would render observed VE as underestimates. Second, JYNNEOS vaccine doses might be undercounted. Reporting doses to NYSIIS was optional before the July 29 state executive order mandated reporting; however, reporting was determined to be mostly complete via inventory surveys.**** Doses administered to New York residents while out of state are not reported to NYSIIS, unless entered afterward by an in-state provider. Both undercounts would cause nondifferential misclassification of coverage, lowering observed VE. Third, as with other studies, it was not possible to account for postvaccination behavior change; however, to the extent that vaccinated case- and control patients became infected in the postvaccination period, observed VE would represent unbiased estimates for those with ongoing risk. Fourth, the outbreak trajectory precluded determining duration of protection. Fifth, data were insufficient to calculate VE by subcutaneous or intradermal administration modes or by HIV-related factors. Finally, the findings describe diagnosed, symptomatic mpox, but not prevention of asymptomatic infection or secondary transmission.

The mpox outbreak rapidly declined during summer 2022 after extensive public health and vaccination efforts and individual behavior changes (*9*). How much decline was attributable to VE, behavior changes, or seasonal variation in viral transmission or behavior is unknown^††††^ (*9*,*10*). Nonetheless, this study leveraged systematically collected patient and vaccine registry data to demonstrate a protective effect of the JYNNEOS vaccine, controlling for outbreak trajectory, among persons with behavioral risk. Global mpox spread continues and might accelerate during summer 2023, given remaining unvaccinated persons with behavioral risk^§§§§^ These findings support recommended 2-dose JYNNEOS vaccination consistent with CDC and NYSDOH guidance.^¶¶¶¶^

**Summary**

**What is already known about this topic?**

The JYNNEOS vaccine was deployed in a national and state vaccination campaign during the 2022 monkeypox (mpox) outbreak. Postexposure prophylaxis and vaccination of persons at highest risk (primarily men who have sex with men) were prioritized. Evidence of vaccine effectiveness (VE) from controlled studies has been limited.

**What is added by this report?**

A comparison of men aged ≥18 years who received a diagnosis of mpox during July 24–October 31 in New York to controls with rectal gonorrhea or primary syphilis, based on systematically collected surveillance data, found adjusted combined 1-dose (received ≥14 days earlier) or 2-dose VE of 75.7%.

**What are the implications for public health practice?**

These findings support recommended 2-dose JYNNEOS vaccination consistent with CDC and New York State Department of Health guidance.

## Footnotes

*
https://www.cdc.gov/poxvirus/mpox/response/2022/world-map.html

†
https://www.health.ny.gov/diseases/communicable/zoonoses/mpox/data/

§ The JYNNEOS vaccine is FDA-licensed for 0.5 mL doses, administered subcutaneously. Beginning August 9, 2022, FDA authorized 0.1 mL intradermal administration as a dose-sparing strategy, based on available evidence. NYSDOH implemented intradermal administration on August 29, 2022, after a brief transition period.

¶
https://www.fda.gov/media/131078/download

**
https://www.hhs.gov/about/news/2022/06/28/hhs-announces-enhanced-strategy-vaccinate-protect-at-risk-individuals-from-current-monkeypox-outbreak.html

††
https://aspr.hhs.gov/SNS/Pages/JYNNEOS-Distribution.aspx

§§
https://regs.health.ny.gov/content/section-210-reporting-cases-or-suspected-cases-or-outbreaks-communicable-disease-physicians

¶¶ Persons reported as male mpox case-patients were presumed to be MSM in this analysis (among 70% of patients with recorded sexual activity within 21 days, 85% reported male or transgender partners). All rectal gonorrhea diagnoses were included (among 63% of patients with partner data, 98% reported male or transgender partners). For primary syphilis, diagnoses were excluded if the person reported no male-to-male sexual contact (84% reported risk factor data).

*** Doses of JYNNEOS vaccine administered in New York outside of NYC are reportable to NYSIIS. Doses given to persons in NYC are reported to the Citywide Immunization Registry, which sends all non-NYC New York residents’ records to NYSIIS.

††† Two separate models considered the main four-level dose classification and one that combined “≥14 days after dose 1” and “dose 2” into a single level.

§§§ STIs detected via asymptomatic screening might have been acquired several weeks or months previously, whereas symptomatic STIs are likely more recently acquired. Thus, persons with STIs detected via asymptomatic screening might be at lower current risk for mpox than are persons with symptomatic STIs. Also, persons with health-seeking behaviors (such as STI screening) might be more likely to receive the JYNNEOS vaccine. Alternatively, because asymptomatic screening is recommended annually for all sexually active MSM and every 3–6 months for those with increased behavioral risk, sampling routinely screening MSM might select for those at elevated risk for mpox. https://www.cdc.gov/std/treatment-guidelines/default.htm

¶¶¶
http://medrxiv.org/content/early/2022/12/14/2022.12.13.22282654.abstract

**** A weekly NYSDOH inventory survey of all counties tracked aggregate JYNNEOS dose administrations and found 4,460 more doses administered than were in NYSIIS in the period before mandatory reporting. These doses account for 15% of doses administered during the study analytic period.

††††
http://medrxiv.org/content/early/2023/02/14/2023.02.10.23285772.abstract

§§§§
https://www.cdc.gov/poxvirus/mpox/response/2022/risk-assessment-of-resurgence.html

¶¶¶¶
https://www.cdc.gov/poxvirus/mpox/vaccines/index.html
